# Balanced crystalloids (RInger’s lactate) versus normal Saline in adults with diabetic Ketoacidosis in the Emergency Department (BRISK-ED): a protocol for a pilot randomized controlled trial

**DOI:** 10.1186/s40814-023-01356-5

**Published:** 2023-07-13

**Authors:** Justin W. Yan, Ahmed Slim, Kristine Van Aarsen, Yun-Hee Choi, Christopher Byrne, Naveen Poonai, Haley Collins, Kristin K. Clemens

**Affiliations:** 1 Division of Emergency Medicine, Department of Medicine, Schulich School of Medicine and Dentistry, Western University, London, ON Canada; 2 Lawson Health Research Institute, London Health Sciences Centre, London, ON Canada; 3 Department of Emergency Medicine, London Health Sciences Centre, London, ON Canada; 4 Department of Epidemiology and Biostatistics, Schulich School of Medicine and Dentistry, Western University, London, ON Canada; 5 Departments of Paediatrics, Medicine, and Epidemiology and Biostatistics, Schulich School of Medicine and Dentistry, Western University, London, ON Canada; 6 Patient partner, London, Canada; 7 Division of Endocrinology and Metabolism, Department of Medicine, Schulich School of Medicine and Dentistry, Western University, London, ON Canada

**Keywords:** Diabetic ketoacidosis, Balanced crystalloids, Normal saline, Emergency department, Pilot study, Randomized controlled trial

## Abstract

**Background:**

Current guidelines for diabetic ketoacidosis (DKA) recommend treatment with normal saline (NS). However, NS, with its high chloride concentrations, may worsen acidosis and contribute to a hyperchloremic metabolic acidosis. Alternatives to NS are balanced crystalloids (e.g. Ringer’s Lactate [RL]) which have chloride concentrations similar to human plasma; therefore, treatment with balanced crystalloids may lead to faster DKA resolution. A recent systematic review and meta-analysis by Catahay et al*.* (2022) demonstrated the need for more blinded, high-quality trials comparing NS versus RL in the treatment of DKA.

**Methods:**

We describe a protocol for BRISK-ED (Balanced crystalloids [RInger’s lactate] versus normal Saline in adults with diabetic Ketoacidosis in the Emergency Department). Our study is a single-centre, triple-blind, pilot randomized controlled trial (RCT) of adults (≥ 18 years) with DKA presenting to an academic tertiary care ED in London, Canada.

Patients with clinical suspicion for DKA will be screened and those found to not meet DKA criteria or have euglycemic DKA will be excluded. We will aim to recruit 52 patients with DKA and will randomize them 1:1 to receive intravenous RL or NS.

The primary feasibility outcome will be recruitment rate, and the primary efficacy outcome will be time elapsed from ED presentation to DKA resolution. Secondary outcomes include time to insulin infusion discontinuation, intensive care unit admission, in-hospital death, and major adverse kidney events within 30 days, defined as a composite of: i) death, ii) new renal replacement therapy, or iii) final serum creatinine ≥ 200% baseline at the earliest of hospital discharge or 30 days after ED presentation. Patients, clinicians, and outcome assessors will be blinded to allocation group. We will follow an intention-to-treat analysis. Gehan-Wilcoxon, Mann–Whitney U, or chi-square tests will be used to compare groups as appropriate.

**Discussion:**

The results of this pilot study will inform the design and feasibility of a full-scale, multicentre RCT to assess fluid choice in adult ED patients with DKA. If proven to demonstrate faster resolution of DKA, administration of balanced crystalloids may replace NS in diabetes treatment guidelines and improve patient and health systems outcomes.

**Trial registration:**

ClinicalTrials.gov, Registration # NCT04926740; Registered June 15, 2021.

**Supplementary Information:**

The online version contains supplementary material available at 10.1186/s40814-023-01356-5.

## Background

Diabetic ketoacidosis (DKA) is an acute, life-threatening complication of diabetes requiring treatment with intravenous (IV) fluid and insulin to correct hyperglycemia and reverse acidosis. Current DKA guidelines recommend normal saline (NS—0.9% sodium chloride) for resuscitation and treatment [1–3]. However, saline’s chloride content is higher than that of human plasma and can cause a hyperchloremic metabolic acidosis, particularly when administered in large volumes (often needed in patients with DKA). Use of saline could subsequently worsen the clinical condition of patients who are already in an acidotic state [4–7].

An alternative to saline is balanced crystalloids (e.g. Ringer’s lactate [RL]) which have chloride concentrations similar to human plasma and it has been hypothesized that use of RL may lead to faster DKA resolution. A recent systematic review and meta-analysis by Catahay et al. 2022 [8] identified three published trials (Van Zyl et al. 2012 [9], Self et al. 2020 [10], Ramanan et al. 2021 [11]) comprising a total of 316 adult patients with DKA who received either saline or balanced crystalloids which assessed for the outcome of DKA resolution. Unfortunately, all three studies had significant methodological limitations, leading to high risk of bias. Van Zyl et al. [9] was the only trial that utilized blinding; the other two were open label, leading to potential bias for participants, personnel, and outcome assessors. The Van Zyl et al. trial was also stopped early before accrual of the planned sample size due to slower than expected enrolment and expiry of study consumables [9]. Compliance to fluid protocols was also not reported in the Van Zyl et al. study and was documented to be low in the Ramanan et al. study [11], resulting in potential contamination bias. Additionally, the Ramanan et al. trial was not powered to detect differences in clinical outcomes and only enrolled patients with severe DKA admitted to intensive care units; thus, the results were considered exploratory and have limited generalizability to the general ED population who may not necessarily have severe DKA. Finally, the Self et al. study [10] was a post-hoc subgroup analysis of completed trials (i.e., SMART [12] and SALT-ED [13]) and power was not prospectively calculated. Due to the existing evidence being from small trials, Catahay et al. [8] “*recommend further investigation into the topic of balanced electrolyte solutions versus isotonic saline in adult DKA patients as there are currently very few clinical trials in publication to conclusively make a decision on the verdict*” of whether or not they result in faster DKA resolution.

Therefore, we present our protocol: Balanced crystalloids (RInger’s lactate) versus normal Saline in adults with diabetic Ketoacidosis in the Emergency Department (BRISK-ED). The goal of the BRISK-ED pilot trial is to assess and evaluate practical and operational considerations in conducting a large multicenter trial of RL versus saline in adults with DKA. Our primary objective is to demonstrate feasibility and that our enrolment processes will lead to sufficient patient recruitment in a timely manner. Our secondary objective is to obtain preliminary data on clinical and safety outcomes. If proven feasible, our pilot study will inform the planning of a future, large-scale, multicentre randomized controlled trial to determine if adult patients who are administered IV RL will have faster DKA resolution without a concomitant increase in adverse outcomes when compared with NS.

## Methods

### Study design and setting

The BRISK-ED trial is a single-centre, triple-blind, pilot randomized controlled trial (RCT) of adults (≥ 18 years) presenting to an academic tertiary care ED with DKA. The study setting is London Health Sciences Centre (LHSC)’s Victoria Campus, an academic tertiary care centre with approximately 90,000 ED visits per year. Situated in London, Ontario, Canada, it is the major referral centre for Southwestern Ontario with a catchment population of over 1.5 million people. The study was approved by Western University’s Health Science Research Ethics Board and is registered with ClinicalTrials.gov (NCT#04926740, registered June 15, 2021). The protocol was developed in accordance with the Recommendations from the Standard Protocol Items: Recommendations for Interventional Trials (SPIRIT) guidelines [14, 15] (Additional file 1) and the Consolidated Standards of Reporting Trials (CONSORT) guidelines for randomized pilot feasibility trials [16].

### Selection of participants

Patients with any clinical suspicion for DKA according to the treating emergency physician will be screened and those found to not meet DKA criteria will be excluded. As there are no definitive criteria for diagnosing DKA [3], we will use the criteria employed by Self et al. [10] and the Diabetes Canada guidelines [3] and include ED patients ≥ 18 years with a clinical diagnosis and laboratory values consistent with DKA, including:

- plasma glucose concentration ≥ 14 mmol/L,
- plasma bicarbonate concentration ≤ 18 mmol/L and/or blood pH ≤ 7.30,
- calculated anion gap > 10 mmol/L, and
- presence of ketones/beta-hydroxybutyrate in serum and/or urine.

We will exclude patients who:

- Are initially seen at another ED and transferred to LHSC for care and/or admission
- Receive > 1L of IV fluid prior to enrolment (e.g. pre-hospital by emergency medical services or while waiting to be seen) as this may cause study contamination. This 1L pre-study cut-off amount was used as an exclusion criterion in the Van Zyl et al. study [9]. Additionally, our regional emergency medical services providers routinely administer up to 1L of pre-hospital IV fluid as part of their treatment protocols.
- Are initially enrolled due to clinical suspicion of DKA based on elevated point-of-care glucose, but ultimately do not meet clinical or laboratory criteria for DKA (e.g. hyperglycemia without acidosis and/or elevated ketones)
- Have euglycemic DKA (generally those on sodium glucose transporter-2 inhibitors)

### Screening, consent, and enrolment

During weekday business hours (Monday-Friday 0700–1700), research staff will screen and identify potentially eligible patients using LHSC’s ED tracking board before approaching the treating physician to confirm eligibility. Outside of regular business hours, physicians may also consent and enrol the patients directly into the study. Because the diagnosis of DKA requires laboratory confirmation, all patients with a point-of-care blood glucose confirming hyperglycemia (≥ 14 mmol/L) will be screened for enrolment as a “possible DKA patient”. If the treating physician agrees that DKA is possible and IV fluid is indicated, they will order the study fluid within our hospital’s electronic medical record’s “DKA PowerPlan” (i.e. standardized order set) or ED physician “Quick Orders” page. Study fluid will be administered to the participant per the randomization protocol after consent is obtained. If patients are initially enrolled but the physician ultimately confirms they do not meet DKA criteria, they will be excluded from the analysis. We will review daily ED visit logs to identify patients missed by our screening process to assess for bias in the enrolled versus missed patients.

We will use an integrated model of consent and will obtain informed verbal consent from all participants or their substitute decision-makers prior to study enrolment. This approach has been approved by our REB as both treatment arms are considered standard of care at our institution. Physicians will first read a script to introduce the study and what participation involves to eligible patients. The patients will be provided a brief letter of information (Additional file 2) and will be asked to provide their verbal consent to participate. The physician will document the patient's verbal consent in their clinical notes and answer any questions the patient may have. To ensure that the research team knows which patients are enrolled in the study, a paper form (Additional file 2) will also be completed by the physician that provides patient information, confirmation of their verbal consent, and the name of the physician who obtained consent. Patients will be able to withdraw their consent at any time.

### Intervention and comparator

Enrolled patients will be randomized 1:1 to receive IV RL (intervention) or NS (comparator). Patients, the clinical team (including all ED physicians, nurses, and any other clinical staff), and outcome assessors will be blinded to allocation group. Pharmacy-prepared kits of 8 × 1L bags of blinded study fluid will be kept in a secure space within the ED. This amount is based on the study by Self et al., where a maximum of 7090 mL was administered to a patient [10]. Once packaged, IV bags are useable for 30 days before expiration. If a kit is opened but not used completely, individual 1L bags may be returned to the pharmacy to save on costs.

The randomization list will be prepared by the pharmacy at LHSC’s Victoria Campus. The pharmacy will prepare an opaque covering over each fluid bag within study kits, which will not be removed during the infusion to maintain blinding. Each opaque-covered bag will be labelled with a kit number and scannable bar code to ensure the patient receives study fluid as ordered which will be entered on their electronic Medication Administration Record. We do not foresee any specific circumstances where unblinding will be necessary, but if needed, the opaque covering may be removed to expose the underlying study fluid bag. At any time, if the treating physician chooses to administer a specific non-study fluid for any reason, they may do so at their discretion.

Rate and amount of study fluid given will be at the treating physician’s (both ED and inpatient, if consulted for admission) discretion. Apart from fluid administered, there will be no other changes to the patient’s clinical care. For example, patients will receive standard DKA treatment which may include insulin, electrolyte replacement, sodium bicarbonate, and/or supportive management. LHSC’s DKA treatment protocol involves hourly point-of-care glucose checks and bloodwork (electrolytes including anion gap, and venous blood gas) every two hours while receiving insulin infusions.

### Outcomes

#### Feasibility outcomes

The primary feasibility outcome is patient recruitment rate over the one-year study period. The target recruitment rate is 41.3% (see Appendix). Although we have not set specific targets for the following, secondary feasibility outcomes we will measure and report include compliance with the study protocol (i.e. patients adhering to their allocated treatment), protocol deviations (i.e. the treating physicians following allocation), frequency of missed eligible patients, the need to break allocation concealment, and loss to follow-up (expected to be negligible due to our outcomes being hospital-based and easily determined).

#### Efficacy outcomes

This pilot is not powered to determine differences in treatment groups; however, a priori outcome definition and accurate outcome assessment is needed to inform the future study. The following efficacy outcomes, consistent with those used by the previous study by Self et al. [10], will also be assessed:

- Primary efficacy outcome: time to DKA resolution (hours), defined as the time elapsed between ED presentation and ketoacidosis resolution, following criteria from the American Diabetes Association Consensus Statement on Hyperglycemic Crises [1] (plasma glucose < 11.1 mmol/L and two of: plasma bicarbonate ≥ 15 mmol/L, venous pH > 7.3 or anion gap ≤ 12 mmol/L).

Of note, Diabetes Canada’s guidelines lack definitive criteria for DKA resolution, only stating that insulin infusion should continue until ketosis resolves (measured by”normalization of plasma anion gap”) [3]. Following the Self et al. study [10], patients discharged prior to evidence of laboratory criteria for DKA resolution will be classified as having DKA resolution at the time of discharge.

- Secondary efficacy outcomes include:
  1. Time to insulin infusion discontinuation (hours)
  2. Intensive care unit admission and length of stay (days)
  3. Total hospital length of stay (days)
  4. In-hospital death
  5. Hyper- or hypokalemia (> 6.0 or < 3.0 mmol/L) post-ED
  6. In-hospital acute kidney injury post-ED (Stage 2 or greater – defined as serum creatinine increase > 200% from baseline or < 0.5 mL/kg/hr urine output for < 12 h)
  7. Major adverse kidney events within 30 days, defined as a composite of: i) death, ii) new renal replacement therapy, or iii) final serum creatinine ≥ 200% baseline at the earliest of hospital discharge or 30 days after ED presentation

We will also collect patient characteristics (e.g. sex, date of birth), the patient’s medical history (e.g. comorbidities, medications), arrival ED information (e.g. Canadian Triage and Acuity Scale score, arrival vital signs), hospital interventions (e.g. IV fluids, medications, supportive management administered), comprehensive laboratory results, and discharge and outcome information (e.g. length of stay, intubation, intensive care unit admission, final diagnoses). All data and outcomes listed above can be ascertained via electronic medical records review, which will occur at 1, 3, 7, and 30 days post-enrolment. Study data for each patient will be abstracted from the hospital’s electronic medical records into a secure, study-specific REDCap data storage platform held at Lawson Health Research Institute. The local research team will have direct access to the study data set.

### Sample size

The sample size for the future trial will be ultimately informed by the recruitment rates and feasibility data obtained in the proposed pilot study; however, we provide a sample size estimation based on the current best available evidence. Based upon our local institutional data and the previously published literature, a full-scale, multi-centered RCT would require 516 participants (258 per arm), assuming α = 0.05, power = 80%, 1:1 allocation, a 40% (6.76 h) minimal clinically important reduction in DKA resolution time (based on expert consensus and patient partner feedback as there is no accepted minimal clinically important reduction in the literature), and 10% attrition rate. This future trial will be conducted at 6 ED sites over 2 years. Based on this, the sample size for this pilot RCT is 52 participants (26 per arm). Appendix provides full detail on our sample size calculation.

### Analysis

We will follow an intention-to-treat analysis. Descriptive statistics will be used to summarize patient characteristics and for the primary feasibility outcome. Comparison of the two allocation groups for the primary outcome of time to DKA resolution will be done using the Gehan-Wilcoxon test because it is a non-parametric test most appropriately used when there is censoring for a survival outcome. Mann–Whitney U tests will compare groups for secondary continuous outcomes including time to insulin infusion discontinuation and hospital length of stay. Finally, chi-square tests will be used to compare groups for categorical variables and 95% confidence intervals will be calculated where appropriate. We do not anticipate missing data for our data variables or outcomes.

A Data and Safety Monitoring Committee consisting of two physicians (e.g. ED, internist, and/or endocrinologist) and a methodologist will review blinded data once 50% of eligible enrolments has been accrued, and will monitor study progress, safeguard data quality, and protect the overall interests of enrolled participants. We will not perform interim analyses for this pilot study.

## Discussion

The BRISK-ED trial will be the second blinded, RCT studying fluid choice in adults with DKA. We will rigorously collect clinical indicators of DKA resolution, patient-important outcomes and adverse events while addressing the limitations and weaknesses of previous studies.

Existing guidelines have highlighted the difficulty of diagnosing DKA, given that there are no clear and definitive diagnostic criteria [3]. A strength of the present protocol involves our use of pre-existing criteria for “DKA” as well as “resolution of DKA” so as to be consistent with previous literature. Additionally, our protocol advises a low screening threshold of suspicion for DKA to enrol a patient into our study in order to minimize missed patients; those who do not prove to have DKA will ultimately be excluded from our analysis.

The success of this protocol relies heavily on established relationships outside of the ED clinical and research teams, as resident and attending physicians representing our hospital’s admitting services will need to continue IV fluid administration for enrolled patients. We have thus planned several mitigating strategies to ensure engagement of key stakeholders in the trial, including: involvement of our study site’s Chief of Medicine as a collaborator, presenting the protocol at departmental rounds, outlining enrolment and study procedures on posters within the ED and on our hospital’s intranet, and embedding the BRISK-ED study fluid orders within our electronic medical record’s “DKA PowerPlan” (i.e., standardized order set) and ED physician “Quick Orders” page. Clinical informatics specialists and pharmacy services will also be an integral part of this protocol to ensure seamless integration of study fluid ordering, availability, and documentation of administration into the patient’s electronic medical record. While these are strategies we have planned locally, similar tailored approaches will be required for future studies at other sites to ensure protocol adherence.

## Conclusions

This study will provide important preliminary and feasibility data to inform a larger definitive trial to evaluate the use of RL compared to NS as part of DKA care in the ED. Ultimately, should a full-scale RCT prove that RL is associated with faster resolution of DKA, administration of balanced crystalloids may replace NS in diabetes treatment guidelines and improve patient and health systems outcomes worldwide.

### Supplementary Information

**Additional file 1. **SPIRIT Checklist.

**Additional file 2. **Letter of information and documentation of consent.

## Data Availability

Not applicable.

## Appendix

### Sample size calculation

The full-scale multi-centre trial will include 516 participants (258 per arm), assuming α = 0.05, power = 80%, 1:1 allocation, a 40% (6.76 h) minimal clinically important reduction in DKA resolution time, and 10% attrition rate. This trial will be conducted at 6 ED sites over 2 years. Based on this, the sample size for this local pilot RCT is 52 participants (26 per arm).

#### Sample size for full-scale trial

The sample size calculation for this trial was based on a study of Clinical Effects of Balanced Crystalloids vs Saline in Adults with Diabetic Ketoacidosis [10] which compared the clinical effects of balanced crystalloids with the clinical effects of saline for the acute treatment in DKA in two clinical trials (Isotonic Solutions and Major Adverse Renal Events Trial [SMART] [12] and the Saline Against Lactated Ringer's or Plasma-Lyte in the Emergency Department [SALT-ED] [13]). The primary outcome for this comparison was the time between ED presentation and DKA resolution, measured in hours. Self et al. (2020) found an absolute reduction in time to DKA resolution of 3.9 h. In the balanced crystalloids group (*n* = 94), the median time to resolution of DKA was 13.0 h [IQR: 9.5–18.8], while in the saline group (*n* = 78) the median time to resolution was 16.9 h [IQR: 11.9–34.5]. The IQR was used to calculate the standard deviation for each group based on the following assumption for normally distributed data: SD = IQR/1.35. The pooled standard deviation was then calculated based on the sample size and standard deviation of each group from the Self et al. (2020) study [√((n1-1)*SD12 + (n2- 1)*SD22)/(n1 + n2-2))] and was determined to be 12.37. To establish superiority of balanced crystalloids versus saline in the time to resolution of DKA, a superiority margin for a clinically significant difference was chosen to be a 40% (= 6.76 h) reduction in time to resolution of DKA based on expert consensus and patient partner feedback. A conservative attrition rate of 10% was selected for the sample size calculation, as loss to follow-up rates should be low given the nature of the intervention (IV fluids) and follow-up period (< 24 h). The actual attrition rate determined by this pilot study will inform the sample size calculation for the full-scale multicentre study. Therefore, to achieve 80% power at the 5% level of significance with equal allocation, the sample size for the balanced crystalloids (Ringer’s lactate) group and the saline group, while accounting for a 10% loss to follow up and a 25% reduction in time to DKA resolution, is 516 participants (258 per group). The sample size was calculated using Wang and Ji's (2020) method [17] for common clinical study designs available at http://riskcalc.org:3838/samplesize/.

We plan to conduct the full-scale trial at 6 ED sites over 2 years, which would require an average minimum recruitment of 86 participants per site (43 per site per year). Our research group has established relationships with these other Canadian EDs where we have previously conducted successful studies. If further sites are needed for recruitment, we will leverage the Network of Canadian Emergency Researchers (NCER).

#### Sample size for pilot trial

For the full-scale trial, a minimum of 43 participants must be recruited annually per site on average. The LHSC Victoria Campus ED treats approximately 130 patients with DKA annually, based on our hospital’s Decision Support data from the most recent fiscal year prior to protocol development (Mar 1 2019 – Feb 29 2020).

**Table:**

DKA by Site	Patients
**Victoria Hospital**	**130**
(E1010) Type 1 DM with ketoacidosis	70
(E1110) Type 2 DM with ketoacidosis	51
(E1112) Type 2 DM with keto & lactic acidosis	1
(E1410) Unspecified DM with ketoacidosis	8

Based on our research team hours of coverage and past data from ED presentation time of potentially eligible patients, we expect to approach at least 104 (80%) of eligible patients in the one-year pilot study period, and a minimum of 43 approached participants (41.3%) must be recruited to meet the feasibility target. According to data from similar past trials, we anticipate being able to recruit at least 50% of approached patients (target sample size of 52 patients, 26 in each arm). With 104 patients approached per year, a 90% two-sided confidence interval around the anticipated recruitment rate will have a total width of 0.17, i.e. a lower limit of 0.415 and an upper limit of 0.585. Because the lower limit excludes the minimum feasibility target of 41.3%, we can be 90% confident that the future trial is feasible.

## Abbreviations

BRISK-ED: Balanced crystalloids (RInger’s lactate) versus normal Saline in adults with diabetic Ketoacidosis in the Emergency Department
CONSORT: Consolidated Standards of Reporting Trials
DKA: Diabetic ketoacidosis
ED: Emergency department
IV: Intravenous
LHSC: London Health Sciences Centre
NS: Normal saline
REDCap: Research Electronic Data Capture
RCT: Randomized controlled trial
RL: Ringer’s Lactate
SGLT-2: Sodium-glucose cotransporter 2
SPIRIT: Standard Protocol Items: Recommendations for Interventional Trials
