# Supplementary material for: Balanced crystalloids (RInger’s lactate) versus normal Saline in adults with diabetic Ketoacidosis in the Emergency Department (BRISK-ED): a protocol for a pilot randomized controlled trial
Source: Pilot Feasibility Stud. 2023 Jul 13;9:121. doi: 10.1186/s40814-023-01356-5 (PMC10339541; doi:10.1186/s40814-023-01356-5)
Supplement: Supplementary file 2 — Additional file 2. Letter of information and documentation of consent. [file 40814_2023_1356_MOESM2_ESM.docx]

**ADDITIONAL FILE 2: LETTER OF INFORMATION AND DOCUMENTATION OF CONSENT**

**
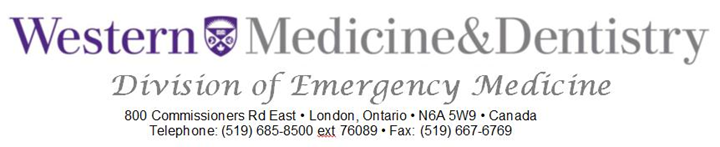
**

**Letter of Information**

**BRISK-ED: Balanced crystalloids (RInger’s lactate) versus normal Saline in adults with diabetic Ketoacidosis in the Emergency Department: a pilot randomized controlled trial**

**Principal Investigator:**

Dr. Justin W. Yan, BSc (Hon), MD, MSc, FRCPC

Assistant Professor, Division of Emergency Medicine

London Health Sciences Centre 519-685-8500 ext. 55014

**Co-Investigators:**

Dr. Christopher Byrne, MD, MSc, FRCPC, Division of Emergency Medicine

Dr. Kristin K. Clemens, MD, Department of Medicine, Division of Endocrinology and Metabolism, Department of Epidemiology and Biostatistics

Dr. Naveen Poonai, MD, MSc, FRCPC, Department of Pediatrics/Medicine (Division of Emergency Medicine)/Epidemiology and Biostatistics

Dr. Yun-Hee Choi, PhD, Department of Epidemiology and Biostatistics

**Research Coordinators:**

Erica Figgins, MSc, Division of Emergency Medicine, London Health Sciences Centre

Branka Vujcic, MSc, Division of Emergency Medicine, London Health Sciences Centre

**Research Assistants:**

Tom Chen, MSc, Division of Emergency Medicine, London Health Sciences Centre

Fardowsa Halane, BSc, Division of Emergency Medicine, London Health Sciences Centre

**Background & Purpose**

You are invited to participate in this study because you have visited the emergency department (ED) with high blood sugar and possible diabetic ketoacidosis (DKA), a condition where the body does not have enough insulin or cannot effectively use the insulin that is produced. As a result, the body produces a chemical called “ketones” as another source of energy, which increase the acid levels of blood and impairs organ function throughout the body. In the emergency department, patients with DKA are usually treated with insulin and large amounts of intravenous fluid. Recent research suggests the fluid type used may be important in treating DKA. Normal saline (0.9% sodium chloride) is the most commonly used intravenous (IV) fluid in treating DKA, but it has a very high concentration of chloride and can lead to additional acid production when given in large volumes. Ringer’s Lactate is another type of intravenous fluid that more closely matches the chemistry of fluid in our bodies and in theory, does not increase the acidity of blood. While there may be benefits to giving Ringer’s Lactate instead of normal saline, past studies have included very few patients and thus, definite recommendations on preferred fluid type still cannot be made. Both fluid types have been shown to be safe when given to patients with high blood sugar.

This letter contains information about the study to help you decide whether or not to participate. Before deciding to participate, please review this letter carefully and do not hesitate to ask questions if there is anything you do not understand.

Our research hopes to find out whether, in adults with DKA, giving Ringer’s Lactate results in faster resolution of DKA compared to normal saline. We hypothesize that patients who are given Ringer’s Lactate will have faster resolution of DKA. If our hypothesis is correct, our results will provide scientific proof that we should change current diabetic ketoacidosis guidelines with respect to fluid choice.

The purpose of this study, called a pilot study or a feasibility study, is to ensure that a larger study will be practical and feasible on a scale involving multiple emergency departments across Canada. This type of study involves a small number of participants and so it is not expected to prove safety or effectiveness. The results may be used as a guide for larger studies, although there is no guarantee that they will be conducted. Completion of a larger study across

multiple sites with more patients will improve our understanding of how fluid choice influences

patient-important outcomes such as faster resolution of DKA (meaning patients can leave

hospital sooner), fewer admissions to the intensive care unit, fewer deaths and fewer cases of

permanent kidney damage. Participation in a pilot study does not mean that you will be eligible to participate in a future larger study.

The overall goal is to determine if Ringer’s Lactate will resolve DKA faster than normal saline. A total of 52 participants (26 per group) will be recruited for this pilot trial at the Victoria Hospital ED, London Health Sciences Centre.

**Study Procedures**

If you choose to enroll in this study, you will be randomly assigned (like a flip of a coin) to 1 of 2 groups by a computer-generated program. You will have a 50/50 chance of being randomized to either receive:

(1) IV Normal Saline

(2) IV Ringer’s Lactate

Neither you, the study staff, nor the study doctors can choose what group you will be in.

The Normal Saline Group will receive the usual standard of care and normal saline intravenous fluid to treat your DKA.

The Ringer’s Lactate Group will receive the usual standard of care and Ringer’s Lactate intravenous fluid to treat your DKA.

We will monitor the time it takes for your DKA to resolve via the bloodwork your healthcare providers order. You will not be asked for any more bloodwork than what is typically needed in a patient with DKA. We will also take note of your disposition after your ED visit (hospital/ICU admission), and hospital length of stay (if admitted).

Apart from the type of IV fluid given, there will be no other changes to your clinical care, and you will receive standard DKA treatment which may include insulin or electrolyte replacement. If your physician thinks you require further tests (e.g. repeated bloodwork, x-rays, etc.) or medications (e.g. insulin, antibiotics, etc.) or admission to hospital, you will continue to receive this care.

For participants in both groups, your electronic medical record on the hospital system (Cerner) will be reviewed at enrollment to collect information related to your ED visit for DKA.

Should you choose to participate, you will be enrolled in the study for the duration of your ED visit. In the event that you are admitted to the hospital during this ED visit, you will continue receiving the same treatment for your DKA that was started in the ED until your DKA resolves.

**Risks & Benefits**

Your participation in this study is entirely voluntary. You may refuse to participate, refuse to answer any questions, or withdraw from the study at any time with no effect on your future care. You do not waive any legal rights by signing the consent form. You will receive a copy of this information letter should you consent to participate in the study. There are no known risks to participating in this study, other than a low possibility of a privacy breach occurring with the data collected during this study. However, the study team will take all necessary precautions to prevent this from happening and will remove any personal identifiers from all data collection forms. If you are randomized to the Ringer’s Lactate Group, there is a possibility that you may benefit from this intervention. The possible benefit you may experience from the administration of the Ringer’s Lactate intravenous fluid may include improved faster resolution of your DKA than if you were to receive normal saline.

However, there is no guarantee that you will benefit personally from participating in this pilot study. This study will contribute important knowledge regarding DKA treatment. This pilot study will directly inform us if a full-scale clinical trial evaluating the use of a Ringer’s Lactate compared to normal saline as part of DKA care in the ED is possible.

**Compensation**

Those who choose to participate in this study will not be compensated.

**Confidentiality**

Your personal health information will be stored confidentially according to standard procedures, with layers of protection in place at the hospital. Any paper research records will be stored in a locked cabinet in a secure office at LHSC, and electronic records will be password-protected on REDCap (secure data collection tool) which is held at Lawson Health Research Institute. They will be viewed only by members of the research team. If the results of this study are published, your name will not be used and no information that discloses your identity will be released or published. Any information connecting your name to your identification will be stored

separately from the data that we collect. Data will be retained for a period of 15 years after publication in a secure place, after which time it will be destroyed in a secure manner (e.g. shredded or electronically destroyed). Qualified representatives of the following organizations may look at the study records at the site where these records are held, for quality assurance (to check that the information collected for the study is correct and follows proper laws and guidelines).

Examples include:

• Representatives of Lawson Quality Assurance Education Program

• Representatives of the University of Western Ontario Health Sciences Research Ethics Board that oversees the ethical conduct of this study.

The study investigators will be permitted to access de-sensitized information only for analysis (i.e., any information that can directly identify a person will be removed or replaced with a code that is not known to the study investigators).

If you have any questions about your rights as a research participant or the conduct of this study, you may contact the Patient Relations Office at LHSC at (519) 685-8500 ext. 52036. If you have specific questions about this study, you may contact the study research coordinator, Branka Vujcic, (branka.vujcic@lhsc.on.ca) at 519-685-8500 ext. 55014.

Thank you,

Dr. Justin Yan, BSc (Hon), MD, MSc, FRCPC

Assistant Professor

Schulich School of Medicine & Dentistry

Western University

**Consent Form**

**Title of Project:** BRISK-ED: Balanced crystalloids (RInger’s Lactate) versus normal Saline in adults with diabetic Ketoacidosis in the Emergency Department: a pilot randomized controlled trial

**Principal Investigator:** Dr. Justin Yan, Emergency Medicine, London Health Sciences Centre

I have read the Letter of Information, have had the nature of the study explained to me and I agree to participate. All questions have been answered to my satisfaction.

___________________ ________________________ ____________

Participant Signature Participant Name (Printed) Date

___________________ ________________________ ___________

Signature of Person Person Obtaining Informed Date

Obtaining Informed Consent Consent (Printed)
